# Attitudes and acceptability of children, caregivers, and healthcare providers about using telemedicine for pediatric HIV care in a resource-limited setting

**DOI:** 10.1371/journal.pone.0268740

**Published:** 2022-05-25

**Authors:** Mamta Manglani, Mamatha Murad Lala, Yashwant Gabhale, Sudha Balakrishnan, Khanindra Bhuyan, B. B. Rewari, Maninder Singh Setia

**Affiliations:** 1 Department of Pediatrics, Pediatric Centre of Excellence for HIV, LTM Medical College and General Hospital, Mumbai, India; 2 UNICEF, New Delhi, India; 3 HIV/STI/HEP WHO, Regional Office for South East Asia, (Formerly, National Program Office, CST, National AIDS Control Organization, India), New Delhi, India; 4 MGM Institute of Health Sciences, Navi Mumbai, India; PLOS ONE, UNITED KINGDOM

## Abstract

**Background:**

The Pediatric HIV Telemedicine Initiative is a video-linked delivery of expert services, designed to reach those previously unable to access expert HIV care. The present qualitative study was designed to understand the acceptability of telemedicine [TM] by patients, their caregivers and health care providers in the anti-retroviral therapy (ART) centers in Maharashtra.

**Methods:**

We conducted focus group discussions with caregivers at six ART centres (three linked with TM facilities and three not linked with TM). We also conducted in-depth interviews with medical officers, counselors, and pharmacists at each centre. The data from the interviews were transcribed and translated into English for analysis. The qualitative data were analyzed using thematic framework approach.

**Results:**

Children and caregivers who had participated in telemedicine consultation through video conference found the process acceptable, were comfortable communicating during these sessions, and did not have any specific problem to report. The advantages of TM were: consultation without having to travel to other cities; economic advantage; and prompt consultation. The total time spent during the process and technical difficulties during the TM sessions were some of the challenges. The medical officers had the opportunity to discuss difficult cases with the expert during the TM session. Some sessions were also considered a ’group counseling’ session, wherein several children and caregivers were able to interact and learn from each other and motivate each other. The health care providers at the three centers that did not currently have TM facilities expressed a desire to have these services at their centers as well. According to them, these facilities will help them address complicated and difficult pediatric HIV cases. Currently, they send their patients to referral centers or other hospitals. Since, many of these referral hospitals are situated in bigger cities, less than 50% of patients access care at these centers This is mostly due to the time constraints and finances (travel/stay) required for accessing these centres.

**Discussion:**

TM was a feasible, acceptable, and desired approach for care of children living with HIV/AIDS. It provides support to their caregivers as well as their care providers. The ART staff from the telemedicine-linked peripheral centers were supportive of the use of TM and wanted these services to be initiated in the non-linked centers.

## Introduction

With advanced and affordable technology making the world more connected, health interventions through novel technological approaches are becoming a reality. The World Health Organization has defined telemedicine (TM) as “*the delivery of health care services*, *where distance is a critical factor*, *by all health care professionals using information and communication technologies for the exchange of valid information for diagnosis*, *treatment and prevention of disease and injuries*, *research and evaluation*, *and for the continuing education of health care providers*, *all in the interest of advancing the health of individuals and their communities”* [1, 2]. It has the potential to improve patient health outcomes through virtual access to health care leading to reduced healthcare costs. As TM applications continue to evolve, it is important to understand its impact on patients, their caretakers and the healthcare professionals. Regardless of the location of the patient, TM connectivity can be harnessed to deliver clinical information, permit consultation and discussion between healthcare professionals and patients, thereby increasing the access to specialist health care that might not otherwise be physically possible. Patients can be monitored more often with TM and appropriate interventions can be delivered in a timely manner. Frequent counseling through TM may motivate patients and their caretakers to become more involved in their own care. All these mechanisms might improve patient health outcomes.

Internationally, the Indian TM pilot projects are largely being viewed as successful. In general, in these pilot projects, patients were satisfied with the care provided through TM [3]. The government funded of HIV treatment began in 2004–05 in India; it was facilitated by the National AIDS Control Organization (NACO) of India through a network of anti-retroviral therapy (ART) centers [4]. The National Paediatric HIV/AIDS Initiative was incorporated into the ART centers in 2006. As of 2019, there were an estimated 2.3 million (1.8–3.1 million) people living with HIV (PLHIV) in India; of these 79,000 are children living with HIV (CLHA) and they are 3.4% of the total PLHIV estimates [5]. However, most ART centres are staffed with general physicians and not by pediatricians or pediatric HIV specialists. Consequently, CLHAs who need expert advice are referred to the nearest ART Plus centers or to the Pediatric Center of Excellence for HIV Care (PCoE). The PCoEs are responsible for care and treatment of children, management of complicated opportunistic infections, specialized laboratory services, training, and research related activities. Furthermore, these centers also provide specific counseling services on nutrition, age appropriate disclosure, and any other specific counseling needs of children and adolescents [6].

The Pediatric Center of Excellence for HIV Care for the state of Maharashtra situated at the Lokmanya Tilak Municipal Medical College & General Hospital, Sion, Mumbai, is one of the seven centers in the country. The distances that the patients are expected to travel from the hard to reach tribal and rural interiors of Maharashtra state to the PCoE at Mumbai for expert opinion and tertiary level treatment can be staggering; it can be as close as 50 km and the farthest distance is about 850 km. Most often, the caretakers are not in a position, physically, financially, or otherwise to forego their daily wages to access services from the far away tertiary center. Thus, these appointments are frequently missed, and the child often deteriorates and dies. To address this issue, the pediatric HIV telemedicine initiative, a e-decentralized model of expert Pediatric HIV care and referral services was established at Lokmanya Tilak Municipal Medical College and General Hospital, Sion, Mumbai (PCoE for the State of Maharashtra) in October 2013. It was started as a multi partnered collaboration between the NACO, India, Maharashtra State AIDS Control Society (MSACS), National Health Mission in Maharashtra (NHM), Municipal Corporation of Greater Mumbai (MCGM) and UNICEF. The state has a high burden of pediatric HIV with an estimate of 8, 940 to 17,440 children living with HIV registered across the 91 ART centers [5].

This pediatric HIV telemedicine initiative is a video-linked delivery of expert services, designed to reach the unreached HIV infected infants, children, and adolescents. These children and their caregivers can access expert care virtually from their parent ART centers without having to travel to Mumbai. The parent ART centres are the centres from where they get their ART treatment regularly; sometimes these centres are in hard to reach tribal and hilly areas of the vast state of Maharashtra. This saves time, effort, and expenses incurred for the reaching the PCoE in Mumbai. Furthermore, promptness of consultation, which can generally be completed within a short period, enables caregivers to ensure that children get timely treatment.

TM consultation through video conference is conducted with the patient and their caretakers along with the team of doctors and counselors treating them. Planned telemedicine sessions are conducted with two ART centres every day for five days in a week; each TM session lasts for two hours. We discuss specific pediatric cases with the doctor or counsellor (based on the requirement); the patient is also present during this consultation. Apart from patient consultations, issues and challenges specific to each centre for overall care of children are also discussed. In addition, need-based TM sessions are conducted; they are done when any ART center requests expert guidance for a complicated pediatric HIV case. Till the time of this publication over 5000 difficult-to-reach pediatric HIV infants, children, and adolescents have received expert medical advice along with counseling through TM. We also conduct group counseling sessions and peer support sessions for multiple patients and caregivers simultaneously; during these sessions patients and caregivers interact and draw support from each other. We also conduct mentoring and capacity building sessions for senior medical officers, medical officers, counselors, data managers, and pharmacists in these ART centres in some of the TM sessions. Some of the topics covered in these mentoring sessions are: paediatric HIV diagnosis and treatment, management of opportunistic infections, management of treatment failures and drug toxicities, monitoring of growth and development, nutrition, immunization, counseling for HIV related issues, counseling for adolescent CLHAs, age appropriate disclosure, and any specific requirement by the ART team. Some of the TM sessions are dedicated for mortality reviews.; thus, we aim to improve the overall capacity of the healthcare providers.

The present qualitative study was designed to study the acceptability and feasibility of TM by the patients, their caregivers and health care providers in addition to the usual HIV care and treatment they continue to receive at the ART centers. We also tried to understand the advantages and problems experienced by those who had used the TM services.

## Methods

This present study is a qualitative health research in six ART centres of Maharashtra; three of these ART centres were linked to the TM facilities and three were not linked to these facilities.

### Data source

We conducted focus group discussions (FGDs) at each of the six centres with children and their caregivers. We conducted FGDs with 27 caregivers at linked ART centres (16 in Bhandara, six in Jalna, and five in Ahmednagar). We conducted FGDs with 21 caregivers at non-linked ART centres (seven in Akola, nine in Pandharpur, and five in Kolhapur). The primary discussion was held with caregivers. However, children were encouraged to share their opinion on certain issues. All the selected ART centres were informed about the FGDs; they were asked to invite children and their caregivers for the interview. Once, they reached the ART centre, we informed them about the FGD. They were told that they have the right to refuse to participate in the FGD. All those who agreed to be a part of the FGD were included in the session. None of those present for the FGD refused to participate in the session. The children had undergone age-appropriate disclosure. The age group of the children were as follows: 1) 1 to 5 years– 15 (31%); 2) 6 to 12 years– 19 (40%); and 3) 13–17 (29%). The gender distribution of the study population was as follows: males (26 [54%]) and females (22 [46%]). There were no significant differences in the age and gender of children across these two types of ART centres (linked and non-linked). About 35 (73%) children were accompanied by their parent (mother or father)), seven (15%) were accompanied by their grandparent, and six were accompanied by other caregivers (other relatives of members of a non-governmental organization). All the children/caregivers who were at the linked centres had participated in a video-conference session.

The FGD guide discussed the following topics: 1) HIV treatment history (duration of follow-up at the ART centre, and treatment/care received); 2) access to various types of services (specifically at the ART centre and in other departments of the hospital in which the ART centre is located); and 3) concerns/satisfaction with the services at the ART centre. These three topics were asked in all the six centres. In the three centres with TM facilities we asked: 1) acceptance of telemedicine consultation through video conference (those who had participated in the same); 2) problems experienced during the process; and 3) suggestions about improving these TM services and other services in general. In the three centres without TM facilities we asked: 1) If TM will be acceptable to them (the questioning started by asking whether they had heard of TM, from whom, had they heard in the context of care of CLHA); and 2) Advantages of having a TM facility at the ART centre (provide some concrete examples where they felt the need for these services).

We conducted in-depth interviews (IDIs) with medical officers, counselors, and pharmacists at each of these six centres. Thus, six interviews from doctors, six from counsellors, and six from pharmacists were included in the present analysis. These interviews were conducted on the same day as the FGD. However, none of these were present in the FGD sessions. They did not have access to the information shared by the caregivers/children in the FGD.

The topics covered in these in-depth interviews were: common clinical complaints and opportunistic infections, concerns about treating CLHA, counseling issues, types of counseling services offered, problems faced by patients (and their caregivers), issues in dispensing and collecting medications (to pharmacists), and potential solutions to challenges that were raised by interviewees. We also discussed the specific role of TM in management of various issues among CLHA at linked centres. In the non-linked centres, we asked about the need for TM connectivity and its potential role in management of CLHA.

### Data transcription and analysis

The FGDs and IDIs were conducted in Hindi, Marathi, or English (based on the language of choice of the participants). The audio files recorded during the interviews were given a unique ID based on the mode of data collection, place of interview, study participants and order of interview, and stored in a secure computer. The data from the interviews were transcribed in the language used by the interviewee. During transcription, any information that could identify an individual was removed and replaced with an alphabetical code to ensure confidentiality and anonymity. The transcribed data were later translated into English for analysis. All the data were analyzed by a single analyst (who has been trained in Qualitative Health Research).

Qualitative data was analyzed using thematic frame work approach [7]. We read the transcript thoroughly and familiarised ourselves with the contents of the transcribed text. We then proceeded with the Thematic Framework. We had identified some a priority themes (such as access to ART services, and advantages and concerns about TM), which were examined in detail in the data. We also defined additional themes according to the issues discussed by the participants. At the end of this process, we had identified concepts in the major themes, and sub-themes. This was followed by indexing of the data. We applied the index systematically to the transcribed textual data and annotated the text with numerical codes. This was followed by charting of the data. We rearranged the indexed data according to the thematic framework. All the related items were placed under appropriate sub-themes. Finally, we used charts for mapping and interpretation, by defining the concepts in the themes, range, and nature of issues covered in various themes and sub-themes. We used NVivo Version 8 (© QSR International), software for the analysis.

The study was approved by the Ethics Committee of Lokmanya Tilak Municipal Medical College and General Hospital (Reference No. IEC57/15). All the participants provided a written informed consent. The caregivers also consented for children (in addition to a verbal assent by children who were above the age of five years).

## Results

We have presented the results according to the themes and sub-themes in our data. The first major part of analysis was about HIV care received in the ART centre. The two main themes in HIV care were access to HIV services and concerns about HIV services received at the centre. The subthemes under concerns about HIV services were: attitude of health care providers; and referral to other health centres. The next part of analysis was on telemedicine services. The main themes in this analysis were: acceptance of TM services; advantages of TM consultations; challenges of TM consultations; and acceptance of TM services among health care providers. The sub-themes under advantages of TM consultations were: consultation without physically travelling to other cities; economic advantages; and prompt consultation. The sub-themes under challenges of TM consultations were: total time spent during the TM process; and technical issues. The two sub-themes under acceptance of TM services among health care providers: opinion about TM in centres that have these facilities; and need for TM in centres that do not have these facilities. We have provided detailed analysis in the next sections.

### I) HIV care

#### I. A. Access to HIV services

The children have accessed services at these centres for varying periods of time. Some of them were attached to the centres from their time of diagnosis while others were referred to these centres from other ART centres.

In general, most of the caregivers were satisfied with the care received at the ART centres. They felt that the health care providers take appropriate care of the children. The counsellors were praised by most of the caregivers. They felt that the counsellors knew the case in detail, they were helpful during the regular and emergency visits, and were able to address most of their health and other concerns.

#### I.B. Concerns about HIV services at the centre

The two major concerns were:

- *Attitudes of health care providers*. Some of the caregivers, however, felt that even though the personnel in the ART centres were non-judgmental, few of the health-care providers in the general hospital and wards were not necessarily sensitive and non-stigmatising. Many HIV infected children are admitted in the paediatric wards (during HIV related or other emergencies and opportunistic infections) and taken care by health care providers not directly attached to the ART centre, and their behaviour directly impacts the care provided to the children and their caregivers. It was suggested that the doctors from the general wards and outpatient departments should also be sensitized for care of CLHAs.
- *Referral to other centers*. If tests are not available in their health care centre (e.g. certain blood tests, radiological examinations), patients are referred to other private centres. Since many caregivers are single parents or grandparents, they have limited resources and are unable to pay for these additional costs. Furthermore, these caregivers already spend to cover travel costs to ART centres (particularly if they come from distant villages). In many instances, additional tests are not conducted due to financial constraints.

*“We spend about 80–100 rupees on bus and other travel to visit the ART centre*. *After that we have to lose our daily wages*. *How will we get the money*?*”*

A caregiver, ART centre

### II) Telemedicine services

#### II.A. Acceptance of TM services

Children who participated in the FGDs had also participated in TM consultations along with the caregivers. These children and caregivers were given an appointment for the scheduled TM consultation. These consultations were conducted in the telemedicine room in the ART center / hospital. They were comfortable communicating via a virtual platform, and were able to discuss their health concerns and problems with the physicians and counsellors present at the PCoE in Mumbai. All the caregivers who had been a part of the telemedicine consultation through video conference found the process acceptable.

The children were also comfortable with the process, and they accepted this form of consultation with doctor. These children found the technological aspect of the consultation extremely attractive. In fact, some of the older children who had been a part of the process found this to be as satisfying as being seen by doctor in the clinic.

*“It is nice to see yourself on the television screen*. *You also get to see the doctor from the other side*.*”*

               - A child who had been a part of a TM session

Caregivers at centres without this facility were aware of these TM consultations. They had heard about TM from various sources (friends, television, advertisements) and were eager to have these technologies at their ART centres as well.

#### II.B. Advantages of telemedicine consultations

*i*. *Consultation without physically travelling to other cities*. Access to expert consultation without having to travel to their city was cited as a big advantage of TM consultation by these caregivers. Many of these care providers are daily wage earners or self-employed in small businesses. Thus, missing their days at work meant loss of income for most of them.

Some caregivers are grandparents. They may not be able to travel to a larger city like Mumbai for consultation. Hence, they find this type of consultation more acceptable.

*“We are old and have no one in Mumbai*. *We cannot take care of ourselves in that city*… *How will we take care of the children*? *Where will we stay*? *How will we get the money for travel and living in Mumbai*? *If there is no teleconsultation*, *we will not be able to take our grandchildren to Mumbai for examination*. *We will have to live with whatever we have*. *What can we do*?*”*

                - A grandmother of a CLHA

*ii*. *Economic advantages*. No additional costs are incurred by the caregivers for these TM consultations. As it is many of them spend money to travel to the ART centres (if they do not stay within the city/area). Thus, travel to Mumbai (including stay) will be an additional expenditure. Many caregivers said that they do not have sufficient resources to cover these additional expenses.

***“**As it is, we spend money to travel to ART centres, care of children… .. How to get more money to go to Mumbai?”*

                - A caregiver at the ART centre

*iii*. *Prompt consultation*. These TM consultations are usually organized within a short time (in case of need based /planned consultation) on the same day of the visit. Therefore, the period between desired consultation and actual consultation is minimized. Many caregivers said that if they were to physically travel for a consultation to Mumbai, they might have to take care of a lot of issues–leave from work / school, additional economic cost of travel, cost of stay in Mumbai, or care of other children who are at home. Thus, some or all these factors may delay their consultation visit with the expert in Mumbai. Hence, they find TM consultations more acceptable than physical visits. This reduces the time for consultation, and saves the cost and effort of travel.

#### II.C. Challenges with telemedicine consultations

Two main challenges were reported regarding the use of TM:

*i*. *Total time spent during the TM process*. Some of the caregivers were concerned about the time spent for the whole teleconsultation process. Although the process itself was not time consuming, they are often called two to three hours before the consultation begins.

*“They call us very early–sometimes at 8*:*00 or 9*:*00 am*. *The teleconsultation starts at 11*:*00 am or later*. *We waste a lot of time waiting for the process to start*. *If you know that the teleconsultation will start at 11*:*00 am; then call us at 11*:*00 am or slightly before 11*:*00*? *We will come*. *But why should we wait unnecessarily*?*”*

                  - A caregiver at an ART centre with TM facility

*ii*. *Technical issues*. Sometimes, there were technical problems in connecting during the teleconsultation process. These were: slow internet speed, lack of connectivity, or power failure. However, these were rare occurrences and not considered to be major impediments in the consultation process.

#### II.D. Acceptance of TM among health care providers

*i*. *Opinion about TM in centres that have these facilities*. All the health care providers (HCPs) had participated in TM sessions. All of them found these sessions to be helpful. The medical officers consider this as an opportunity to discuss difficult cases with the doctors at PCoE. They usually discuss two to three cases per session. They present the cases and discuss the clinical issues of the new cases, as well as follow-up findings/reports of previously discussed cases (such as inadequate weight gain, opportunistic infections, change of regime etc.) during these sessions. Furthermore, the counselors discuss counseling issues (such as unsatisfactory weight gain, nutrition counselling, adherence counseling, importance of growth monitoring, timely immunizations, early detection of opportunistic infections, psycho-social coping aspects, etc.) with the consultant at PCoE during TM sessions.

*“I may not be able to pinpoint one case which has benefited from the VC sessions*, *but I know that*, *overall*, *our management of children infected with HIV has improved due to these sessions*. *We discuss nutrition*, *drug adherence*, *and social problems of these children”*

                  -Medical Officer in a TM Centre

The medical officers also found the presence of counselor and nutritionist in the TM sessions particularly helpful. They can discuss all aspects of the case during these sessions. This helps them understand other aspects of care (such as counseling and nutritional aspects).

*“One good thing about the VC session is that the counselor from the PCoE is always present*. *That is immensely helpful for my counselors*. *They learn various aspects of counselling during these sessions”*

               - Medical Officer in a TM Centre

The counselors also found these sessions extremely useful. They can discuss counselling issues and problems with CLHAs during these sessions. Furthermore, some of the counsellors felt that patients are more likely to adhere to the advice given during these sessions. If the PCoE team counsels them about adherence, nutrition and care of children, the caregivers are more likely to adhere to this advice. The counselor at the Civil Hospitals can then follow-up with these children and caregivers during the regular follow-up sessions.

Another counselor stated that a major advantage of the TM session was its role as a “group counseling” session. It was highlighted that in some sessions, there are many children and caregivers during the VC session. In these sessions, if the PCoE consultant or counselor addresses the concern of one child or caregiver, others can hear the solution and implement them if it is applicable to them. Furthermore, if other children or caregivers face similar problems, they vocalize these during the sessions. Thus, TM can be used for ‘group counseling’ regularly both for children and their caregivers.

The doctors and counselors also said that they will face a lot of problems, if the TM facilities were stopped. In such a scenario, they will have to refer the patients to referral hospitals, or to the PCoE at Mumbai. Many of the referral centres are far and the caregivers will probably not take the children to these centres. Thus, they will miss the advanced care that they would have received in the TM session.

*ii*. *Need for TM facilities in the centres that do not have facilities*. The doctors and counselors at the three centres that did not have TM facilities also expressed a desire to have these services at their centres. These services will help them with care of complicated cases and difficult cases in the centre itself. Currently, they refer these patients to referral centres or other hospitals. Many of these referral hospitals are situated in bigger cities. The doctors said that less than 50% of patients that are referred to these centers are able to access care at these centers due to time/ financial constraints. With TM facilities, they will be able to discuss these difficult cases with experts in the PCoE.

Most of the ART centres that do not have TM facilities are in medical colleges. It was suggested that since these centres have specialists and specialty departments, the complicated cases can be handled in these colleges. However, as highlighted in the in-depth interview with the medical officer in one centre, we found that even though these cases were referred to paediatricians in the hospital, the paediatricians usually consulted back with the medical officers in the ART centres, due to inadequate knowledge and lack of experience of management of CLHAs.

*“We refer these children to paediatricians*. *However*, *they consult us back for ART related issues*… . *Hence*, *telemedicine facilities will be useful for us*. *Right now*, *we discuss these cases with other doctors who are a part of our phone group”*

                - Medical Officer in an ART centre with no TM facility

Many of the paediatricians working in the medical colleges lack experience in treating CLHA. Even though these ART centers are in medical colleges, they should be connected with TM services.

Some of the caregivers in these centres had heard about TM facilities. However, others had not. When we explained the concept to them, they seemed very eager about having these facilities in their parent ART centres. According to them, it will be useful to avoid travel to other far-off places. It can be difficult to go to these referral hospitals with children. Sometimes both the parents take the child to these referral centres. Even if the clinical examination at the referral centres does not cost, travelling to these centres is expensive and daily wage earners lose income for the days of travel. Furthermore, they are in unfamiliar surroundings and find it difficult to manage in these settings.

*“If we have such facilities in our centre*, *it will be useful*. *We can talk to the expert directly*. *You know how difficult it is to travel with a child in the crowd”*

         - A parent at one of the ART centres which did not have TM facility

*“Of course, these services will be important and useful. Usually, they will refer us to a Civil Hospital. It is not always convenient for the patient to go to the Civil Hospital. You have to reach there on time—before 9 am. It so difficult to reach there and finish everything in one day since it takes time for examination and investigations. The patients usually have no place to stay. We have already come here. So, if the expert pediatrician can do it while we are here….it will be nice*.

         - A parent at one of the ART centres which did not have TM facility

Thus, the health care providers and caregivers believed having TM facilities in their centres will be extremely useful.

We have also presented all the quotes in Table 1.

**Table 1:** Table of quotations from the qualitative study on the use of telemedicine (TM) for children living with HIV/AIDS (CLHA), Maharashtra, India.

Quotation List	Actual Quotation	Participant
Quotation 1	*“We spend about 80–100 rupees on bus and other travel to visit the ART centre*. *After that we have to lose our daily wages*. *How will we get the money*?*”*	A caregiver, ART centre
Quotation 2	*“It is nice to see yourself on the television screen*. *You also get to see the doctor from the other side*.*”*	A child who had been a part of a TM session
Quotation 3	*“We are old and have no one in Mumbai*. *We can’t take care of ourselves in that city*.* *.* *. *How will we take care of the children*? *Where will we stay*? *How will we get the money for travel and living in Mumbai*? *If there is no teleconsultation we will not be able to take our grandchildren to Mumbai for examination*. *We will have to live with whatever we have*. *What can we do*?*”*	A grandmother of a CLHA
Quotation 4	***“****As it is we spend money to travel to ART centres*, *care of children*.* *.* *.* *.. *How to get more money to go to Mumbai*?*”*	A caregiver at the ART centre
Quotation 5	*“They call us very early–sometimes at 8*:*00 or 9*:*00 am*. *The telemedicine starts at 11*:*00 am or later*. *We waste a lot of time waiting for the telemedicine process to start*. *If you know that the teleconsultation will start at 11*:*00 am; then call us at 11*:*00 am or slightly before 11*:*00*? *We will come*. *But why should we wait unnecessarily*?*”*	A caregiver at an ART centre with TM facility
Quotation 6	*“I may not be able to pin-point one case which has benefitted from the VC sessions*, *but I know that*, *overall*, *our management of children infected with HIV has improved due to these sessions*. *We discuss nutrition*, *drug adherence*, *and social problems with these children”*	Medical Officer in a TM Centre
Quotation 7	*“One good thing about the VC session is that the counselor from the PCoE is always present*. *That is immensely helpful for my counselors*. *They learn various aspects of counselling during these sessions”*	Medical Officer in a TM Centre
Quotation 8	*“We refer these children to paediatricians*. *However*, *they consult us back for ART related issues*.* *.* *.* *. *Hence*, *telemedicine facilities will be useful for us*. *Right now*, *we discuss these cases with other doctors who are a part of our phone group”*	Medical Officer in an ART centre with no TM facility
Quotation 9	*“If we have such facilities in our centre*, *it will be useful*. *We can talk to the expert directly*. *You know how difficult it is to travel with a child in the crowd”*	A parent at one of the ART centres which did not have TM facility
Quotation 10	*“Of course*, *these services will be important and useful*. *Usually*, *they will refer us to a Civil Hospital*. *It is not always convenient for the patient to go to the Civil Hospital*. *You have to reach there on time—before 9 am*. *It so difficult to reach there and finish everything in one day since it takes time for examination and investigations*. *The patients usually have no place to stay*. *We have already come here*. *So*, *if the expert pediatrician can do it while we are here…*.*it will be nice*.	A parent at one of the ART centres which did not have TM facility

## Discussion

The present qualitative study provides useful information on the role of TM in providing HIV services for the pediatric population in India. Overall, it was acceptable by children, their caregivers, and doctors. Even though, some of the common complaints of excess time spent at the ART centres and technical problems were reported, all of them found the service extremely useful. It saves travel time and money spent to visit specialized centres. Even though TM services were initiated in centres not attached to teaching hospitals, however, we found that teaching hospitals and medical colleges also need expertise for care of HIV infected children. Hence, extension of these TM services should be considered for all health care centres in the State.

There is a large urban-rural disparity in the health care facilities in India; most of the facilities that have advanced services are in urban areas [8]. Consequently, patients are often referred to other centres for expert opinion (diagnostic or therapeutic). This adds to patient burden of the already overburdened tertiary care centres, particularly in urban areas. Implementation of telemedicine has been beneficial for rural health and primary health care in India [9, 10]. Studies have shown that caregivers avail of TM options and these tele-health services reduces family care giver burden [11, 12]. As seen in our study, only about 50% of children who are referred for expert HIV care actually avail of the services. This may be due to economic reasons (lack of money, loss of working days, grandparents as caregivers, or not willing to go to the city if they do not know anyone). Thus, TM services not only will improve the referral of CLHAs but also reduce the burden on tertiary care centres. Furthermore, it is also important to upscale investigation facilities in rural centres and ensure that all necessary tests are available on-site. If the tests/investigations are not available, then it will be useful to develop links with local laboratories and imaging centres. Prompt investigations will help in TM consultation as well as effective management of CLHAs.

Several reviews, evaluating different types of TM technologies and telephone consultations, have been published. Overall, research has established that there are high levels of patient satisfaction associated with TM as a result of saving travel cost, waiting time, and easy accessibility of specialists [13]. In fact, a systematic review found that the effectiveness of the TM may depend on multiple factors–such as the nature and severity of the disease, trajectory of the medical condition, the nature of services (clinical, monitoring, or diagnostic services), the health care provider and the health care system involved in providing the services [14]. Our TM services were for a chronic condition which requires specialized training for the care of the population. Even though infectious disease specialists may be comfortable in handling adult HIV cases, pediatric HIV care may require additional expertise. Furthermore, our study found that these services were appreciated by caregivers across a variety of health care centres (such as district hospitals or medical colleges). Finally, other studies have shown that primary care physicians who access TM services reported higher confidence in care of HIV infected individuals and improved management of cases [15, 16]. Thus, medical officers in rural and semi-urban areas in Maharashtra may become confident in care of HIV infected children, due to these TM services.

TM has been used for infectious diseases and HIV subspecialty care, even in hard-to-reach areas such as prisons and correctional facilities [17–19]. In our study, we were able to provide acceptable and specialized services over a large geographic area. Another advantage of the TM is the group counseling sessions. Though, there may be concerns about confidentiality, only consenting children and their caregivers attend these sessions. The session usually becomes a useful exercise for all the attendees. Each member learns from others, some questions are common, whereas some concerns are expressed by more than one caregiver. The solutions provided by the health care provider or even the attendee may be useful for everyone. Furthermore, as reported in our findings children also enjoy seeing themselves on the screen. Thus, medical care becomes slightly more interesting compared with the mundane hospital check-up. Furthermore, even the physicians reported that overall HIV care of the pediatric population had improved in their centres, even though concerns about technological aspects of telemedicine (such as slow internet and power failure) was important–an issue that has been highlighted as an important challenge for TM by health care providers [20]. With the government focused on providing digital services in the country [21], this TM initiative can be considered a model-project and may be emulated in other states to provide specialized care to CLHA.

In the present manuscript, we have not presented clinical parameters as the focus of this study was to understand the acceptability and feasibility of TM for HIV care in the pediatric population. However, it was a part of a broader study to assess the effectiveness of TM in care of CLHA in Maharashtra. We did find that CLHA in the linked centres were more likely to be alive and the proportion of children who were ‘lost to follow-up’ was lower in the linked centres compared with non-linked centres [22]. Furthermore, we have also compared the clinical management of children in linked centres and non-linked centres (these six centres) [23]. We found that complete physical examination, tuberculosis screening, and co-trimoxazole prophylaxis was significantly more likely in CLHAs in the linked centres compared with non-linked centres [23]. In addition, CLHAs in linked centres were more likely to be investigated and managed appropriately compared with non-linked centres [23]. Other studies have also shown that TM facilities had better health outcomes [19, 24, 25]. In summary, the qualitative findings from our study suggest that TM consultation has been a feasible, acceptable, and desired approach for management of CLHAs in semi-urban and rural parts. It also provides support for their caregivers as well as their care providers. The ART staff from the telemedicine-linked peripheral centers were also supportive of the use of TM and wanted these services to not only continue in their centers, but also be initiated in the non-linked centers.

### Conclusions

TM has been proven to be useful in a number of specialties, and will also be successful in the context of HIV/AIDS, as several factors such as stigma, fear, and discrimination are all possibly negated to a certain extent in the virtual medium. The key findings in our qualitative study showed that the TM initiative for reaching out to the difficult-to-reach HIV infected infants, children and adolescents was effective, acceptable, and feasible for the patients, their caregivers as well as their health care providers. TM was found to be helpful for medical officers and counselors in addressing difficult complicated cases. TM improved geographical and financial access to paediatric HIV care, with significant cost savings both from the provider side and the patient side [26]. Health care providers were also incredibly supportive of the use of TM and wanted these services to not only continue in their centers, but also be initiated in the non-linked centers.
